# Preventing Silicone Tube Extrusion after Nasolacrimal Duct Intubation in Children

**Published:** 2010-10

**Authors:** Ali-Akbar Sabermoghaddam, Setareh Sagheb Hosseinpoor

**Affiliations:** Eye Research Center, Mashhad University of Medical Sciences, Mashhad, Iran

**Keywords:** Nasolacrimal Duct Intubation, Silicone Tube Extrusion, Crawford Tube, Nasolacrimal Duct Obstruction

## Abstract

Herein we report our experience with a simple technique for reducing the rate of silicone tube extrusion after nasolacrimal duct (NLD) intubation for congenital NLD obstruction. Medical records of children older than 2 years, with or without history of failed probing, who had undergone NLD intubation with a Crawford silicone tube over a period of 4 years were reviewed. In all subjects, one end of the Crawford tube was passed through a piece of scalp vein tubing followed by applying one or two knots. All Crawford tubes were removed after 3 months. Main outcome measures included complications such as tube extrusion, nasal discharge, crust formation and pyogenic granuloma formation. Fifty-seven patients, including 49 unilateral and 8 bilateral cases with mean age of 3.8±1.6 (range, 2 to 11.5) years were operated. No complications such as tube dislodgement, significant nasal discharge, crust or pyogenic granuloma formation occurred prior to Crawford tube removal. All silicone tubes were successfully removed from the nasal cavity. In conclusion, passing one end of the Crawford tube through a small piece of scalp vein tubing before knotting it in the nasal cavity seems to decrease the rate of tube extrusion which is the most common complication following NLD intubation in children.

## INTRODUCTION

**Figure 1:**
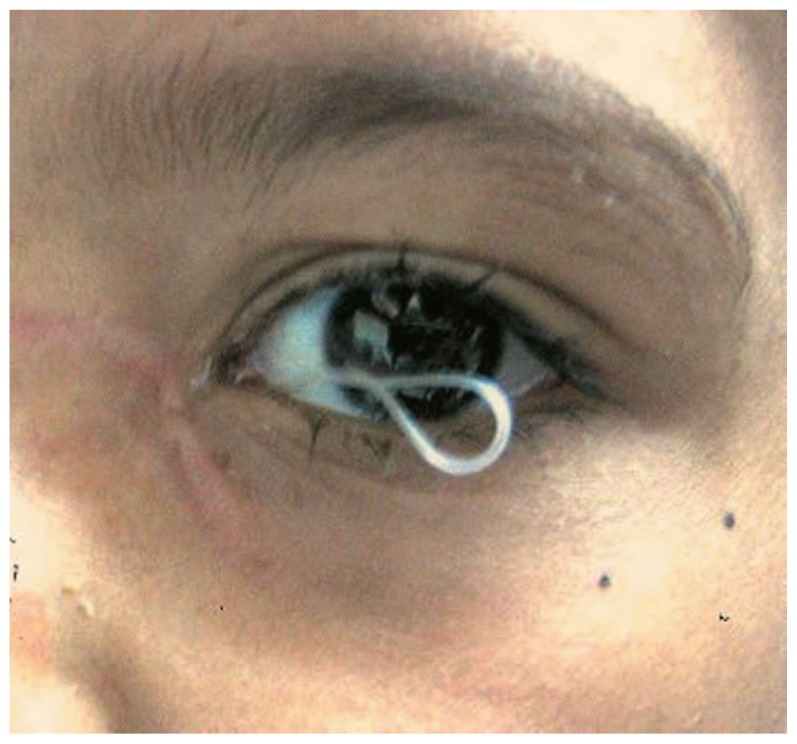
Tube extrusion with corneal irrigation and discharge.

Congenital nasolacrimal duct (NLD) obstruction occurs in 1.2% to 20% of newborns.^1^ In 90% of cases the obstruction resolves within 6 months with conservative measures including massage, maintaining eyelid hygiene and topical antibiotics.^2^,^3^ If the condition does not respond to observation, probing is the next option. The success rate of probing decreases with increasing patient age.^4^ Although probing is still successful in older children^5^,^6^, there are many surgeons who choose silicone intubation as the first procedure in older children or as the next step if simple probing has already failed.^7^ The success rate of intubation following failed probing has been reported from 69% to 100% in different studies.^8^–^18^ Intubation may entail complications such as pyogenic granuloma formation, corneal irritation and tube extrusion (Fig. 1); the latter necessitates earlier than planned tube removal.^10^ In this study, we report a simple modification in surgical technique to decrease the rate of tube extrusion which at the same time facilitates its removal.

## SURGICAL TECHNIQUE

**Figure 2:**
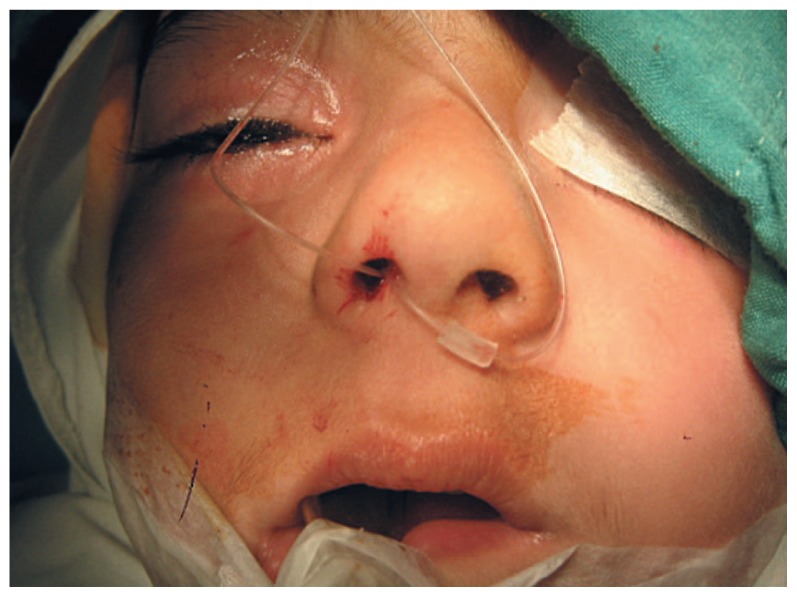
Before applying the knot, one end of the silicone tube was passed through a small piece of scalp vein tubing.

**Figure 3:**
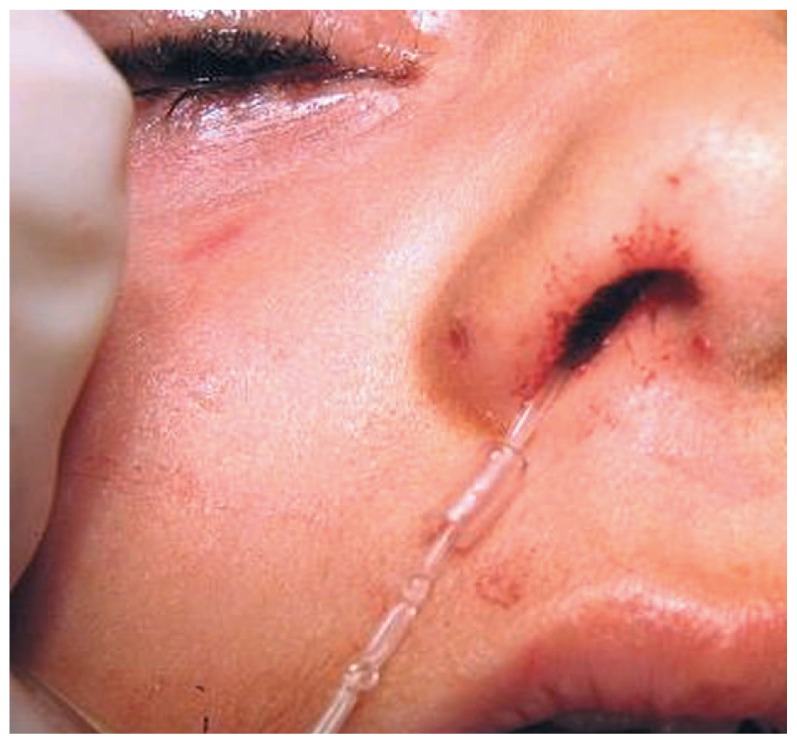
For final fixation, two knots were tied over the scalp vein tube.

We retrospectively reviewed the medical records of children older than 2 years, with or without history of probing, who had undergone nasolacrimal duct (NLD) intubation with a Crawford tube in the operating room. After retrieving the two ends of the tube from the nasal cavity, one end was passed through a small piece (2 to 3 mm) of the silicone tubing of a scalp vein set (Fig. 2); the two ends were then tied together with only one or two knots (Fig. 3). No other technique, such as fixation to the nasal mucosa was used. We encountered no major complications intraoperatively.

Overall, 57 patients (65 eyes) including 30 boys and 27 girls with mean age of 3.8±1.6 (range, 2 to 11.5) years were operated. Demographic data are shown in table 1. All subjects were followed for 3 months at the time which the Crawford tube was removed. At least three months of follow-up was completed for all cases. No complications such as pyogenic granuloma formation, punctal or canalicular damage, crusting or nasal discharge, tube extrusion, or corneal abrasion were observed.

## DISCUSSION

The success rate of silicone intubation for congenital NLD obstruction varies from 50% to 100% in different studies.^8^–^20^ This study is unique in its perspective of managing tube-related problems with a simple modification in surgical technique.

**Figure 4:**
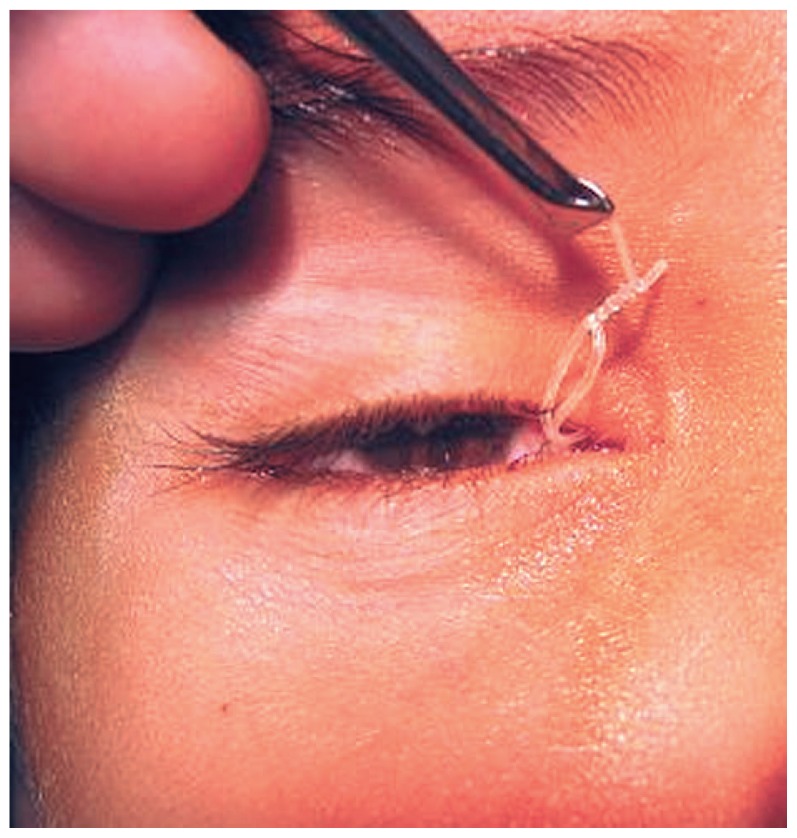
Using the usual technique and despite applying multiple knots, this tube was extruded from the canaliculus.

Silicone intubation was first described by Gibbs^21^. The recommended time for tube removal is 6 weeks to 18 months postoperatively.^20^ However, the tube may require earlier removal because of complications such as extrusion or punctal cheesewiring and dislodgement. In some cases, tube dislodgement may necessitate early removal of the tube. Peterson et al^22^, reported a trend toward persistent epiphora with premature silicone tube removal, however this correlation failed to reach statistical significance. Less favorable outcomes of NLD intubation in children over 2 years of age have been reported in other studies^20^,^23^. Surgeons may decide to change the surgical procedure in these children to ensure tube stability and prevent premature displacement or choose another approach such as balloon dacryoplasty. The rate of complications varies in different studies.^4^,^23^ The most common is tube dislodgement which may be due to tying only one small knot.^12^,^22^ Several techniques have been used to solve this problem such as intranasal fixation of the tube (which entails disadvantages such as increased complexity and surgical time, irritation of the nasal mucosa, formation of pyogenic granulomas and problems in tube removal), using multiple silicone tube knots, and proximal fixation by intrasac or transsac fixation sutures.^11^,^12^,^20^,^24^ However, extrusion of the tube may occur with such methods (Fig. 4). The rate of tube extrusion is higher in the first 3 months following surgery, especially in the first month.^4^ Because of difficulty in repositioning the extruded tube in these young patients, most of them are removed at the time of displacement, before the recommended time for removal. In the current study, we observed no case of tube extrusion during the follow up period. Other complications such as nasal crusting and discharge, corneal abrasions, or congestion of the conjunctiva did not occur in any of our patients.

**Figure 5:**
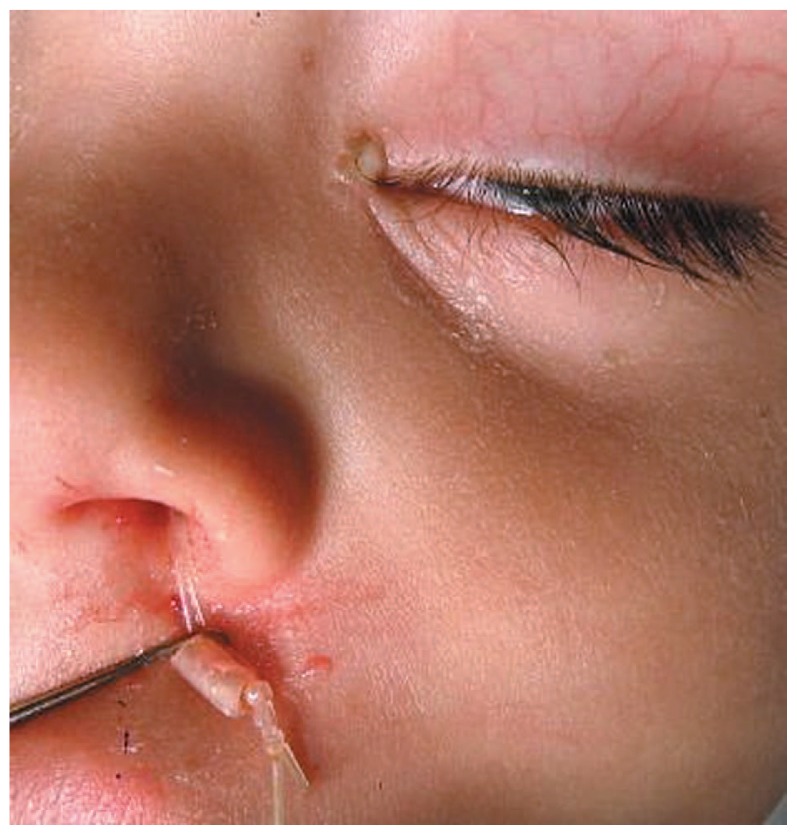
Silicone tube removal seems to be easier using the scalp vein tube.

In summary, we may conclude that passing one end of the Crawford tube through a small piece (2 to 3 mm) of the silicone tubing of a scalp vein set in the nasal cavity may decrease the rate of tube dislodgement and circumvent complications related to other techniques of intranasal silicone tube fixation for NLD intubation. This modification also seems to facilitate locating the nasal end of the silicone tube at the time of removal (Fig. 5).

## Figures and Tables

**Table 1:** Patient demographics

Number of Patients	57

Number of Eyes	65

Sex	
Male	30

Female	27

Mean Age (years)	
Male	3.9 ± 1.4

Female	3.7 ± 1.8

All	3.8 ± 1.6

Involved Eye	
Right	28

Left	21

Bilateral	8
